# Assessment of the Carcinogenic Potential of Pretomanid in Transgenic Tg.rasH2 Mice

**DOI:** 10.1177/10915818221113295

**Published:** 2022-07-18

**Authors:** Jeffrey L. Ambroso, John Dillberger, Rebecca Bruning-Barry, Tian Yang

**Affiliations:** 1 6856 RTI international, RTP, NC, USA; 2 J.Dillberger LLC, Nashville, IN, USA; 3 486654 TB Alliance, New York, NY, USA

**Keywords:** PA-824, genotoxicity, carcinogenicity, nitroheterocyclic compound, Tg.rasH2 mice

## Abstract

Pretomanid is a nitroimidazooxazine antimycobacterial drug that was approved as part of a three-drug oral regimen, consisting of bedaquiline, pretomanid, and linezolid, for 6-months treatment of adults with pulmonary extensively drug-resistant tuberculosis or with complicated forms of multidrug-resistant tuberculosis by the food and drug administration in the United States and regulatory bodies in over 10 other countries. Nitroaromatic compounds as a class carry a risk of genotoxicity and potential carcinogenicity based on reactive metabolite formation. A battery of good laboratory practice genotoxicity studies on pretomanid indicated that the compound was not genotoxic, however its hydroxy imidazole metabolite (M50) was genotoxic in the Ames assay. To assess the in vivo carcinogenic potential of pretomanid, hemizygous Tg.rasH2 mice were administered pretomanid once daily by oral gavage for 26 weeks. Male mice were given pretomanid in vehicle at doses of 0, 5, 15 and 40 mg/kg/day and female mice were given pretomanid in vehicle at doses of 0, 10, 30 and 80 mg/kg/day. Positive control mice of both sexes received intraperitoneal injections of urethane at 1000 mg/kg on Days 1, 3 and 5. There were no pretomanid-related early deaths, tumors, non-neoplastic microscopic findings, or gross necropsy findings at any dose level. The positive control gave the anticipated response of lung tumors. Oral administration of pretomanid to mice produced plasma exposure to the parent compound (high dose AUC of pretomanid 3 times the clinical AUC at the maximum recommended human dose) and exposure to the M50 metabolite (less than 10% of pretomanid) at all dose levels in both sexes. These data show that pretomanid was not carcinogenic in a transgenic mouse model at systemic exposures greater than human therapeutic exposures.

## Introduction

Pretomanid (also known as PA-824, CAS 187235-37-6) is a nitroimidazooxazine antimycobacterial drug with a complex mechanism of action that involves inhibition of cell wall mycolic acid biosynthesis under replicating (aerobic) conditions and respiratory poisoning through generation of reactive nitrogen species, including nitric oxide (NO), under non-replicating (anaerobic) conditions.^1-3^

Pretomanid was recently approved to be marketed in the US and 10 other countries. It is indicated for the treatment of adults with pulmonary extensively drug-resistant tuberculosis or with complicated forms of multidrug-resistant tuberculosis as part of an oral, 6-month, three-drug regimen containing bedaquiline, pretomanid and linezolid. Because pretomanid is taken for 6 months, rodent carcinogenicity studies are part of the expected nonclinical safety package for this drug.^4^

As a class, heterocyclic nitro compounds are considered potentially mutagenic and carcinogenic by way of metabolic activation that can produce reactive oxygen species (ROS) and free radicals.^5^ But not all heterocyclic nitro compounds have produced positive results in mutagenicity and rodent carcinogenicity studies, and the efficacy of these compounds, particularly in the treatment of parasitic diseases, has kept these compounds in the pharmaceutical research pipeline.

This publication reports the results of genotoxicity studies with pretomanid and one of its metabolites (designated as M50) and a 26-week carcinogenicity study in Tg.rasH2 transgenic mice given daily oral doses of pretomanid. Metabolite M50 was evaluated because it is quantifiable in human plasma following administration of pretomanid.

Tg.rasH2 mice carry the human protooncogene c-Ha-ras and develop a high incidence of spontaneous tumors, notably pulmonary tumors, and hemangiosarcomas. This mouse strain has been well-studied and is sensitive to both genotoxic and nongenotoxic carcinogens. A 26-week study in Tg.rasH2 mice has been found to be as useful as a traditional 2-year (lifetime) study in wild-type mice for detecting the carcinogenic potential of known human carcinogens.^6-8^ The FDA believes the Tg.rasH2 mouse model has been adequately evaluated as an alternative model for carcinogenicity testing of pharmaceutical candidates.^6^

## Materials and Methods

### Genetic Toxicity Studies

**Figure 1.**
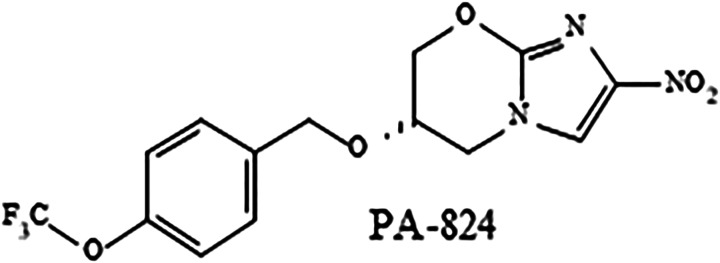
Structure of pretomanid.

**Figure 2.**
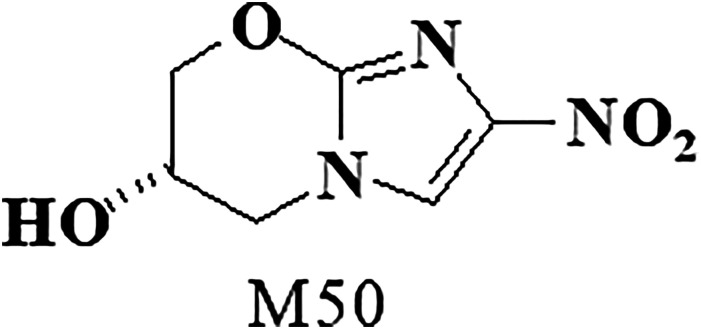
Structure of metabolite M50.

Pretomanid and a metabolite designated as M50 (Figure 1 and Figure 2) were evaluated for mutagenic potential in bacterial reverse mutation assays. Pretomanid also was evaluated for clastogenic potential in a chromosome aberration assay in Chinese hamster ovary (CHO) cells and in a micronucleus assay in erythrocytes harvested from the bone marrow of mice given oral doses. All assays were conducted in compliance with Good Laboratory Practice (GLP) Regulations except the assays conducted with M50.

#### Bacterial Reverse Mutation Assays

Pretomanid and metabolite M50 were evaluated over a range of concentrations, with and without an added metabolic activation system, in five tester strains: *Salmonella typhimurium* histidine auxotrophs TA98, TA100, TA1535, and TA1537^9^ and the *Escherichia coli* tryptophan auxotroph WP2*uvr*A.^10^ *S. typhimurium* strains were received from Dr. Bruce Ames, Department of Biochemistry, University of California (Berkeley, CA). The *E. coli* strain was received from The National Collection of Industrial Bacteria, Torrey Research Station, Aberdeen, Scotland (United Kingdom). The metabolic activation system was a liver homogenate (S9) mix. The S9 component was prepared from male Sprague-Dawley rats that had been injected (intraperitoneally) with Aroclor^TM^ 1254 at 500 mg/kg, 5 days before sacrifice.^9^ The S9 mix was prepared immediately before use and contained 10% S9, 5 mM glucose-6-phosphate, 4 mM β-nicotinamide adenine dinucleotide phosphate, 8 mM MgCl_2_ and 33 mM KCl in a 100 mM phosphate buffer at pH 7.4. Pretomanid was tested at 0.05 to 5000 μg/plate. Metabolite M50 was tested at 78 to 2500 μg/plate. A negative (solvent) control culture and appropriate positive control cultures were included in each assay.

Bacteria were exposed to test materials by the plate incorporation method originally described by Ames et al^9^ and updated by Maron and Ames.^11^ Bottom agar was Vogel-Bonner minimal medium E^12^ with 1.5% (w/v) agar, and the top Agar was 0.7% (w/v) agar and 0.5% (w/v) NaCl. After incubation, cultures were evaluated using a dissecting microscope for precipitation or cytotoxicity, and revertant colonies were counted. The mean numbers of revertant colonies in various cultures were compared to reach conclusions about assay validity and mutagenic potential. For the test article to be evaluated positive, it must cause a dose-related increase in the mean revertants per plate of at least one tester strain with a minimum of two increasing concentrations of test article. Data for strains TA1535 and 1537 were judged positive if the increase in mean revertants at the peak of the dose response was equal to or greater than three times the mean vehicle control value. Data for strains TA98, TA100, and WP2 *uvr*A were judged positive if the increase in mean revertants at the peak of the dose response was equal to or greater than two times the mean vehicle control value.

#### Chromosome Aberration Assay in CHO Cells

CHO-K_1_ cells (American Type Culture Collection) were exposed to pretomanid at 22.5 to 360 μg/mL for 4 hours with and without added S9 mix and for 20 hours without added S9 mix. All cells were harvested 20 hours after treatment initiation. The assay was performed using standard procedures,^13^ including duplicate cultures and appropriate negative and positive controls. Under each set of conditions, three test concentrations were evaluated. The highest was the lowest precipitating concentration, and two additional lower concentrations also were evaluated.

After incubation, cells were harvested and placed on slides, which were examined microscopically to determine relative mitotic index and to evaluate metaphase spreads for structural or numerical chromosome aberrations. Where possible, 200 metaphase spreads were evaluated. The mean numbers of aberrations in various cultures were compared to reach conclusions about assay validity and clastogenic potential.

#### Bone Marrow Micronucleus Assay in Mice

Groups of male CD-1®(ICR) BR mice were given single oral doses of vehicle (0.4% CMC), positive control article, or pretomanid at 125, 500 or 2000 mg/kg and then were euthanized approximately 24 or 48 hours later to harvest femoral bone marrow. Bone marrow was mounted on slides and stained with May-Grünwald solution and Giemsa. For each mouse, 500 red blood cells were examined to determine the ratio of immature red blood cells (polychromatophilic erythrocytes or PCEs) to mature red blood cells (normochromatophilic erythrocytes of NCEs) and 2000 PCEs were examined to determine the ratio of micronucleated PCEs (mnPCEs) to normal PCEs.

The mean PCE-to-NCE ratios in various groups were compared to reach conclusions about assay validity and effects on erythropoiesis. The mean mnPCE-to-PCE ratios in various groups were compared to reach conclusions about assay validity and clastogenicity. Comparisons were made using an analysis of variance on untransformed mnPCE-to-PCE ratios for each mouse and on untransformed PCE-to-NCE ratios when the variances were homogeneous. Ranked proportions were used for heterogeneous variances. If the analysis of variance was statistically significant (*P* ≤ 0.05), Dunnett’s t-test was used to determine which dose groups were statistically significantly different from the vehicle control. Comparisons were made at the 5% probability level.

### Mouse Carcinogenicity Study

#### Test Article, Vehicle, and Positive Control Formulations

Pretomanid (6S)-2-nitro-6-{[4-(trifluoromethoxy)benzyl]oxy}-6,7-dihydro- 5H-imidazo[2,1-b][1,3]oxazine was synthesized by Mayne Pharma Inc (≥99.6% purity by HPLC area%). The vehicle was 0.5% carboxymethylcellulose (CMC) with 0.1% Tween 80 (v/v). The vehicle and pretomanid formulations were administered to mice by oral gavage once daily at a dosing volume of 10 mL/kg. Individual dose volumes were calculated based on each animal’s most recently recorded body weight. The dose formulations were magnetically stirred at room temperature for at least 30 minutes prior to and during dosing. The positive control was urethane in 0.9% sodium chloride and was administered via intraperitoneal injection for a total of three times. The positive control formulation was allowed to stand at room temperature for at least 30 minutes and inverted several times prior to dose administration. Aliquots (0.5 to 1.0 mL) were collected from the top, bottom, and middle of the first (week 1), interim (week 13), and last (week 25) pretomanid dose formulations to assess the accuracy of the concentration and/or homogeneity.

#### Animals

Male and female Tg.rasH2 [CByB6F1-Tg(HRAS)2Jic (+/−hemizygous c-Ha-ras)] mice (n = 110/sex) approximately 5 weeks of age were assigned to the main cohort groups to assess carcinogenicity. Male and female wild type CByB6F1 mice [CByB6F1-Tg(HRAS)2Jic (−/−homozygous c-Ha-ras)] (n = 34/sex) approximately 6 weeks of age were assigned to the cohort of mice used to assess the toxicokinetics of pretomanid and its M50 metabolite. Extra mice were received to ensure the animals were healthy and did not exceed ±20% of the sex-specific group mean weight at randomization on Day 1 of the study. One toxicokinetic (TK) male mouse was outside the specified weight range but was included in the study. All mice were obtained from Taconic Biosciences (Germantown, NY). The supplier verified that the mice were murine pathogen-free and females were nulliparous and non-pregnant. At the time of dosing initiation (Day 1), the toxicokinetic mice were approximately 8 weeks of age. On Day 1 of the study, the body weights of the main cohort mice ranged from 19.2 to 25.9 grams (males) and from 15.9 to 20.4 grams (females), and the body weights of the TK cohort mice ranged from 23.4 to 30.2 grams (males) and from 17.6 to 23.5 grams (females).

During acclimation mice were pair-housed and then moved to individual housing post randomization. Mice were housed in polycarbonate cages under environmentally controlled conditions of temperature, relative humidity, ventilation, and illumination. The mice were acclimatized to the laboratory conditions for approximately 2 weeks before study start. Mice were provided tap water and TEKLAD Global Diet #2018 CM (Certified 18% Protein Rodent Diet, Envigo, Madison, WI) in meal form *ad libitum* until study termination and were provided environmental enrichment, such as nestlets. The water met U.S. EPA drinking water standards. Annual water analyses, individual feed lot analysis, and enrichment materials did not reveal any known contaminants that would have had an effect on the results of this study.

The number of animals, animal procedures, and experimental design for this study were reviewed and approved by the BioReliance (Rockville, MD) Institutional Animal Care and Use Committee. All animal experiments were performed in an Association for Assessment and Accreditation of Laboratory Animal Care (AAALAC)-accredited facility and all procedures involving animals performed followed the specifications recommended in the most current version of The Guide for the Care and Use of Laboratory Animals. (National Academy Press, Washington, D.C.).

#### Experimental Design

This study assessed the carcinogenic potential of pretomanid following repeated oral gavage administration once daily for 26 weeks in hemizygous Tg.rasH2 [CByB6F1-Tg(HRAS)2Jic (+/− hemizygous c-Ha-ras)] mice. This study also evaluated the TK profile of pretomanid and metabolite M50 in wild-type CByB6F1 [CByB6F1-Tg(HRAS)2Jic (−/−homozygous c-Ha-ras)] mice. The study was conducted under GLP regulations.

The protocol was submitted to the U.S. FDA Executive Carcinogenicity Assessment Committee for review and their suggestions incorporated into the final study design. The Tg.rasH2 mouse model is recommended for genotoxic and non-genotoxic carcinogen identification. This short-term (26-Week) carcinogenicity assay is an acceptable alternative to the traditional two-year mouse carcinogenicity assay as described in the ICH S1B guidance.^8^ CByB6F1 mice are the wild-type littermates of the Tg.rasH2 mice and thus have the same genetic background, except for the omission of the Tg element. For this reason, the non-transgenic wild-type littermates were considered acceptable for use in the TK cohort. Dose levels for this study were selected based on a preliminary 28-day study dose range-finding study in CByB6F1 mice (data not shown).

An overview of the experimental design is presented in Table 1. For carcinogenicity assessment (main cohort), 25 mice/sex were included in the vehicle control and pretomanid-treated groups. At the time of dosing initiation (Day 1), the main cohort mice used for assessing carcinogenicity were approximately 7 weeks of age and weighed between 15.9 to 20.4 g (females) and 19.2 to 25.9 g (males). Male groups (Groups 2, 4, and 6) received pretomanid in vehicle at doses of 5, 15, and 40 mg/kg/day, respectively. Female groups (Groups 3, 5, and 7) received pretomanid in vehicle at doses of 10, 30, and 80 mg/kg/day. Positive control mice (n = 10/sex) were included in the main cohort and received three intraperitoneal injections of urethane in saline (1000 mg/kg/injection) on Days 1, 3, and 5. The test and control articles were administered at a dose volume of 10 mL/kg body weight.

**Table 1.** Overview of the Experimental Design for the 26-Week Carcinogenicity Study of Pretomanid in Tg.rasH2 Transgenic Mice.

Group	Dose levels (mg/kg/day)	Number of animals			
		Main cohort (Tg.rasH2)		TK cohort (CByB6F1)^a^	
		Male	Female	Male	Female
1 (vehicle control)	0	25	25	4	4
2	5	25	0	10	0
3	10	0	25	0	10
4	15	25	0	10	0
5	30	0	25	0	10
6	40	25	0	10	0
7	80	0	25	0	10
8 (positive control)	1000 (urethane)	10	10	0	0
Total		110	110	34	34

^a^Extra TK animals (1/sex/group) were used to provide sufficient animals for blood collection in the event of intercurrent deaths.

TK animals were included for the vehicle control (n = 4 sex) and pretomanid-treated groups (n = 10 sex/group). The TK cohort animals were dosed by oral gavage once daily for 14 consecutive days.

The animals were randomized by body weight, using a computer-generated randomization program into dose groups as described in the Table 1 below.

#### In-life Observations and Measurements

Clinical signs, body weights, and food consumption were assessed for the main cohort animals to monitor animal health and any potential toxicity from pretomanid administration. In-life measurements were recorded in Provantis™ Version 9.4.6.3 (Instem, Philadelphia, PA).

All animals, including those in the TK cohort, were observed twice daily for morbidity and mortality. For animals in the main cohort, cage side observations were performed on the day of dosing within 2 hours after the last animal in each group was dosed (Days 1, 3, and 5 for positive control animals). Detailed, hands-on observations were performed on Day 1 and weekly for the main cohort animals.

Body weight measurements were recorded prior to dosing, on Day 1, and then weekly through week 13, switching to biweekly from weeks 13 through 26. Body weights of TK cohort animals were recorded pre-dose on Day 1, 8 and 13. Body weights for TK animals were collected for dose volume calculations only. Food consumption was measured weekly beginning on Day 1 for the main cohort animals.

#### Bioanalysis and Toxicokinetic Assessment

Toxicokinetic blood samples (300 μL for males and 250 μL for females) were collected in K_3_EDTA tubes after 14 doses from the TK cohort via retro-orbital bleeding under 70% CO2/30% O2 anesthesia. On Day 14, control mice were bled once 2 hours after dosing. On Day 14 and 15, Pretomanid-treated mice were bled predose, 0.5, 1, 3, 6, and 24 hours post dose. TK animals were euthanized after their final blood collection.

Collected blood was stored on wet ice until the samples were processed to plasma. Blood samples were centrifuged at 4±2°C and 8000 g for 5 minutes. Plasma was snap frozen in liquid nitrogen and stored in a deep freezer (≤−60°C) within 60 minutes of bleeding the TK animals.

Pretomanid and the M50 metabolite were quantified in mouse plasma by liquid chromatography-tandem mass spectrometry (LC-MS/MS) after protein precipitation. Aliquots of 25 µL of plasma were spiked with internal standards (PA-824-d5 or morphine) and proteins were precipitated in 1% formic acid in acetonitrile. A partial supernatant from each sample was transferred to a clean plate and diluted in acetonitrile/water (40:60) for pretomanid and deionized water for M50. Supernatant was analyzed for pretomanid or M50 using validated LC-MS/MS methods with a linear range of 20 to 10,000 ng/mL for pretomanid and 10 to 5,000 ng/mL for M50 (methods validated at Alliance Pharma, Malvern PA).

Sample collection time and plasma concentration data were analyzed to derive TK parameters by standard model independent methods based on the mean plasma concentration-time data for each dose group. The TK analyses of the plasma concentration profiles were performed using non-compartmental analysis in Phoenix WinNonlin Professional 7.0 (Certara, L.P., St. Louis, MO, USA). Concentration values below the lower limit of quantitation were treated as zeros for analysis purposes. The following parameters and constants were determined: maximal plasma concentration (C_max_), time to maximum plasma concentration (T_max_), area under the plasma concentration-time curve to twenty-four hours postdose (AUC_0-24h_), and terminal elimination half-life (t_1/2_).

#### Postmortem Assessments

Animals that died early during the dosing period were refrigerated as soon as possible (within 8 hours) until necropsied. All animals found dead were verified for death by lack of respiration and toe pinch reflex before being moved to the refrigerator or transferred to necropsy. A full necropsy was performed, and protocol-specified tissues were preserved and evaluated microscopically. One TK animal was evaluated by necropsy to assess for gavage error, but no tissues were preserved.

Full necropsies were performed for vehicle control or pretomanid-treated main cohort animals at the end of the dosing phase (Day 183 or 184). Mice were weighed prior to necropsy then euthanized by IACUC and veterinary-approved procedures that included CO_2_ overdose. The necropsy procedure was a thorough and systemic examination and dissection of the animal viscera and carcass and recorded on a validated, computerized Data Capture System (Provantis Version 9.4.6.3). Mice in the positive control group were euthanized by CO_2_ overdose on Day 71 (females) and 73 (males). Standard tissues in accordance with the ICH and FDA guidelines, were collected for the vehicle control and pretomanid-treated main cohort animals and saved in 10% neutral buffered formalin (NBF) at the time of necropsy with the exception of eyes and testes, which were fixed in Davidson’s fixative and modified Davidson’s fixative, respectively. The positive control animals were subjected to a limited necropsy where only the lungs were collected and saved in 10% NBF; no gross lesions, except for those in the lungs, were recorded.

Animals in the TK cohort were euthanized by CO_2_ overdose as soon as possible after TK blood collection and were not necropsied.

#### Histopathology

Tissues from the main cohort animals, as well as the lungs from all positive control animals, were processed, embedded in paraffin, sectioned, stained with hematoxylin and eosin, and evaluated microscopically. Any remaining fixed tissues were archived per protocol. The microscopic evaluation data reflects a consensus between the study pathologist and a peer review pathologist.

## Statistical Analyses

### In-Life Data

Pretomanid-related changes were assessed by comparing to the vehicle control group. The incidence of all effects was analyzed by sex and dose. Dunnett’s Test was conducted on body weight, body weight changes and food consumption data. Statistical analysis of body weight and food consumption data from positive control animals were not conducted. All required statistics were based on a significance value of *P* ˂ 0.05.

## Survival Data

Tumor pathology and mortality data were transferred to SAS transport format. Statistical analysis of survival and tumor data was conducted by BioSTAT Consultants (Portage, MI), using methods outlined in Refs. 14-16.

Kaplan-Meier estimates of group survival rates were calculated by sex. The generalized Wilcoxon test for survival was used to compare the homogeneity of survival rates across the vehicle control and test article groups by sex. If the survival rates were significantly different (*P* < 0.05), the generalized Wilcoxon test was used to make pairwise comparisons of each test article group with the vehicle control group. Additionally, the positive control group was compared to the vehicle control group using the generalized Wilcoxon test.

Survival times in which the status of the animal’s death is classified as an accidental death, planned interim sacrifice or terminal sacrifice were considered censored values for the purpose of the Kaplan-Meier estimates and survival rate analyses.

## Tumor Data

### Vehicle and Test Article Groups

The incidence of tumors was analyzed by Peto’s mortality-prevalence method,^17^ without continuity correction, incorporating the context (incidental, fatal, or mortality independent) in which tumors were observed. Because of the sparse number of deaths during the study, the following fixed intervals were used for incidental tumor analyses: Days 1 through 120 and Days 121 through and including terminal sacrifice. A minimum exposure of 121 days was considered sufficient to be included with animals surviving through scheduled termination. All tumors in the scheduled terminal sacrifice interval were considered incidental for the purpose of statistical analysis. Tumors classified as mortality-independent were analyzed with Peto’s mortality independent method incorporating the day of detection.

Each diagnosed tumor type was analyzed separately, and analysis of combined tumor types were performed. All metastases and invasive tumors were considered secondary and not statistically analyzed.

A 1-sided comparison of each test article group with the vehicle control was performed. An exact permutation test was conducted for analyses with low tumor incidence. Findings were evaluated for statistical significance at both the 0.01 and 0.05 levels.

### Vehicle and Positive Control Groups

Tumor incidence in the positive control group was compared to the vehicle control group with a 1-sided Fisher’s exact test at both the 0.01 and 0.05 significance levels. Only the following tumors were statistically analyzed: alveolar-bronchiolar adenoma and alveolar-bronchiolar carcinoma.

## Results

### Bacterial Reverse Mutation Assays

Ames assay results are summarized in Table 2. Pretomanid was evaluated at up to 5000 μg/plate in two assays, one using *S. typhimurium* tester strain TA100 and another using *S. typhimurium* tester strains TA98, TA1535, TA1537 and *E.coli* tester strain WP2 *uvrA*. In both assays, precipitate was observed at concentrations ≥1000 μg/plate, but there was no appreciable cytotoxicity. Each assay was considered valid because the vehicle and positive controls gave the expected results. Exposure to pretomanid did not cause a significant increase in the number of revertant colonies in any tester strain under any conditions. Therefore, pretomanid was considered negative for mutagenic potential.

**Table 2.** Ames Assay Results (Mean Number of Revertants/Plate) for Pretomanid and Metabolite M50.

Pretomanid	TA98		TA100		TA1535		TA1537		WP2uvrA	
Conc. (μg/plate)	+S9	−S9	+S9	−S9	+S9	−S9	+S9	−S9	+S9	−S9
Vehicle	24	20	143	98	13	12	7	6	14	16
100	22	25	137	119	12	9	9	5	16	23
333	26	12	179	107	13	10	6	5	17	21
1000	23	18	173	115	10	10	5	10	15	17
3333	16	17	166	99	11	8	5	5	10	11
5000	22	15	179	106	14	7	4	5	14	16
M50	TA98		TA100		TA1535		TA1537		WP2uvrA	
Conc. (μg/plate)	+S9	−S9	+S9	−S9	+S9	−S9	+S9	−S9	+S9	−S9
Vehicle	20	17	108	95	11	13	11	6	17	17
78	18	13	132	120	12	20	9	8	18	18
156	19	16	125	135	15	16	12	13	17	24
313	22	14	252	203	31	19	8	7	23	26
625	25	17	312	294	42	24	14	13	48	29
1250	24	21	666	588	67	43	20	10	50	49
2500	38	33	758	868	81	60	11	18	50	71

Metabolite M50 was evaluated at up to 2500 μg/plate in a single assay. There was no precipitate or cytotoxicity at any concentration. The assay was considered valid because the vehicle and positive controls gave the expected results. Exposure to M50 resulted in significant increases in revertant colonies of *S. typhimurium* strains TA100 and TA1535 and *E. coli* strain WP2uvrA in the presence and absence of S9, in *S. typhimurium* strain TA98 in the presence of S9, and in *S. typhimurium* strain TA1537 in the absence of S9. Therefore, metabolite M50 was considered positive for mutagenic potential.

### Chromosome Aberration Assay in CHO Cells

Precipitate was visible in treatment medium at 360 μg/mL, but no cytotoxicity was observed. The assay was considered valid because the vehicle and positive controls gave the expected results. Exposure to pretomanid did not significantly increase the frequency of chromosome aberrations at any concentration (*P*>0.05). Therefore, pretomanid was considered negative for clastogenic potential in mammalian cells *in vitro*.

### Mouse Bone Marrow Micronucleus Assay

Pretomanid did not induce signs of toxicity in mice given single doses at up to 2000 mg/kg, which is the limit dose based on regulatory guidance contemporary with the time of study conduct. In addition, pretomanid did not affect erythropoiesis at any dose level, ie, it did not produce a statistically significant decrease in the PCE:NCE ratio. Plasma exposure (C_max_ and AUC) to pretomanid increased less than proportionally with dose level. At 2000 mg/kg, the mean C_max_ and AUC_last_ values were 36 μg/mL and 1137 μg*h/mL, respectively. These data confirmed exposure of bone marrow to pretomanid in this study.

The assay was considered valid because the negative and positive controls performed as expected. Pretomanid did not significantly increase the mnPCE-to-PCE ratio at any dose level. Therefore, pretomanid was considered negative for clastogenic potential *in vivo*.

### Carcinogenicity Study in Transgenic Mice

#### Dose Formulation Analysis

Mean measured pretomanid concentrations were 87% to 103% of target, the relative standard deviation in samples from the top, middle, and bottom was ≤ 10.0%, and pretomanid was not detected in the vehicle. Therefore, formulations were accurately prepared and homogenous.

### Mortality

Survival rates did not differ significantly among groups. Survival rates for the vehicle control and pretomanid-treated groups at the low-, mid-, and high-dose levels were 96%, 100%, 100%, and 100%, respectively, for males and 100%, 96%, 100%, and 92%, respectively, for females. Five animals died prior to the scheduled termination and are described in Table 3.

**Table 3.** Summary of Early Deaths.

Group/Sex	Dose (mg/kg/d)	Day of death	Cause of death	Interpretation
1M	0	177	Spinal cord hemagiosarcoma	Natural death
3F	10	184	Multicentric hemangiosarcoma	Natural death
5F	30	7	Caught in feeder	Accidental
7F	80	145	Multicentric lymphoma	Natural death
7F	80	64	Undetermined	Natural death

### Clinical Observations

Pretomanid did not produce clinical signs of toxicity at any dose level. One high dose female had ruffled fur and rapid and shallow breathing and was cold to the touch on Days 139 and 140 and had ruffled fur on Day 144 but this was not considered related to pretomanid treatment.

The positive control article produced ataxia and decreased motor activity, which was expected based on the toxicity of urethane.

### Body Weights

Mice gained weight over the course of the study, and pretomanid did not affect mean body weight at any dose level. The mid dose males had transiently lower body weight gain and the high dose females had transiently higher body weight gain but overall body weight gains for the entire study were similar between the control and pretomanid-treated groups (Tables 4 and 5).

**Table 4.** Mean Male Body Weights (Grams ± SD).

	Study day						
Dose (mg/kg/day)	1	29	57	85	113	141	183
0	21.88 (1.39)	23.57 (1.38)	25.83 (1.50)	27.01 (2.06)	28.63 (2.56)	29.62 (2.74)	31.08 (3.13)
5	22.07 (1.39)	24.02 (1.19)	26.47 (1.45)	27.81 (2.11)	29.42 (2.79)	30.87 (3.12)	32.52 (3.41)
15	22.03 (1.52)	23.82 (1.58)	25.84 (1.84)	27.24 (2.24)	28.67 (2.73)	30.14 (3.03)	31.41 (3.48)
40	21.60 (1.64)	23.44 (1.43)	25.33 (1.49)	26.30 (1.84)	27.63 (2.37)	28.54 (2.62)	30.42 (2.91)
Positive control	21.99 (0.71)	24.81 (1.41)	27.73 (1.74)	NA	NA	NA	NA

Abbreviations: NA, Not assessed as positive control animals were terminated on Day 73.

**Table 5.** Mean Female Body Weights (Grams ± SD).

	Study day						
Dose (mg/kg/day)	1	29	57	85	113	141	183
0	18.31 (0.98)	19.74 (0.86)	21.12 (0.87)	21.77 (1.03)	22.42 (1.38)	22.70 (1.37)	23.80 (1.41)
10	18.10 (0.99)	19.30 (1.00)	20.86 (0.94)	21.52 (1.12)	22.72 (1.29)	22.90 (1.54)	24.08 (1.67)
30	18.51 (0.85)	20.13 (1.00)	21.44 (0.77)	22.02 (1.03)	22.81 (0.99)	23.40 (1.54)	24.29 (1.75)
80	18.27 (0.96)	19.63 (0.94)	21.55 (1.12)	22.05 (1.61)	22.90 (1.90)	23.55 (2.23)	24.63 (4.22)
Positive control	17.89 (0.57)	19.81 (0.58)	22.11 (0.68)	NA	NA	NA	NA

Abbreviations: NA, Not assessed as positive control animals were terminated on Day 71.

The positive control article produced weight loss during the first week of the study (not shown), which was expected based on the toxicity of urethane.

### Food Consumption

Pretomanid did not affect food consumption at any dose level (Tables 6 and 7). The females demonstrated a number of statistically significant increases in mean food consumption spread throughout the study, mostly at 30 mg/kg/day; also, the mean food consumption from Day 1 to 183 was significantly increased at 30 mg/kg/day compared to the vehicle control. These differences in the food consumption were considered incidental to pretomanid treatment due to and their sporadic occurrence and the lack of a dose response.

**Table 6.** Mean Male Food Consumption (Grams/Day ± SD).

	Study days						
Dose (mg/kg/day)	1-8	29-36	57-64	85-92	113-120	141-148	176-183
0	3.610 (0.369)	3.595 (0.473)	3.423 (0.420)	3.653 (0.316)	3.738 (0.459)	3.863 (0.537)	3.830 (0.686)
5	3.960 (0.629)	3.450 (0.211)	3.381 (0.449)	3.726 (0.279)	3.706 (0.308)	3.789 (0.321)	3.705 (0.375)
15	3.914 (0.544)	4.046 (1.140)	3.651 (0.704)	3.922 (0.570)	3.782 (0.548)	3.833 (0.348)	3.838 (0.730)
40	3.966 (0.722)	3.795 (0.471)	3.558 (0.535)	3.855 (0.645)	3.768 (0.487)	3.814 (0.280)	3.980 (0.749)
Positive control	2.894 (0.461)	4.044 (0.374)	3.956 (0.359)	NA	NA	NA	NA

Abbreviations: NA, Not assessed as positive control animals were terminated on Day 71.

**Table 7.** Female Mean Food Consumption (Grams/Day ± SD).

	Study days						
Dose (mg/kg/day)	1-8	29-36	57-64	85-92	113-120	141-148	176-183
0	3.594 (0.565)	4.458 (0.867)	4.228 (0.788)	4.460 (1.015)	4.958 (1.524)	4.550 (1.031)	5.203 (1.511)
10	3.644 (0.610)	4.501 (1.178)	4.179 (0.908)	4.371 (0.800)	5.603 (0.911)	4.441 (0.704)	5.004 (1.343)
30	3.991 (0.514)	4.686 (1.110)	4.513 (0.695)	5.557* (1.179)	5.717 (1.446)	5.540* (1.335)	5.521 (1.116)
80	3.907 (0.699)	4.936 (1.235)	4.697 (0.760)	5.063 (0.730)	6.051* (1.297)	5.353 (1.206)	5.475 (1.178)
Positive control	3.313 (1.048)	6.218 (1.222)	4.210 (0.524)	NA	NA	NA	NA

Abbreviations: NA, Not assessed as positive control animals were terminated on Day 71.

### Neoplastic Microscopic Findings

#### Lung Tumors

Pretomanid did not significantly increase the incidence of lung tumors at any dose level. The incidence of single adenomas, multiple adenomas and/or carcinomas in both sexes and the combined incidence of all lung tumors were similar in the vehicle control and pretomanid-treated mice. All lung tumors in the pretomanid-treated mice fell within the historical control range of the laboratory.

The incidence of alveolar bronchiolar adenomas in vehicle control males (7/25; 28%) slightly exceeded the historical control range (up to 24%) of the laboratory. This difference was considered incidental and did not interfere with data interpretation.

All positive control mice of each sex had alveolar bronchiolar adenomas, and this incidence (100%) was significantly greater (*P* < 0.01) than vehicle control males (28%) and females (4%), establishing the validity of the study. Incidence data for alveolar bronchiolar tumors (adenoma and carcinoma) are provided in Table 8 below.

**Table 8.** Neoplastic Microscopic Findings.

Incidence of Tumors in Males						
Tumor type	Vehicle control (n = 25)	Pretomanid (mg/kg/day)			Positive control (n = 4)	HCR (n = 25-30)
		5 (n = 25)	15 (n = 25)	40 (n = 25)		
**Lung tumors**						
Adenoma						
Single	28%	20%	8%	4%	0%	Up to 24%
Multiple	0%	0%	0%	0%	100%*	Up to 8%
Carcinoma	0%	0%	0%	0%	0%	Up to 12%
Combined	28%	20%	8%	4%	100%*	NA
**Hemangiosarcoma**						
Spleen	12%	0%	0%	12%		0-16%
Kidney	0%	4%	0%	0%		0-4%
Spinal cord, lumbar	4%	0%	0%	0%		0-4%
Combined	16%	4%	0%	12%		NA
**Other tumors**						
Harderian gland adenoma	0%	4%	0%	0%		0-8%
Harderian gland carcinoma	0%	0%	0%	4%		0-12%

*Statistically significant (*P* < 0.01).

Vehicle = 0.5% sodium carboxymethylcellulose/0.1% Tween 80, in deionized water.

Positive control = Three IP injections of 1000 mg/kg/day urethane in 0.9% saline.

HCR = BioReliance Historical Control Range for control mice (25-30 animals/sex/group).

NA = Not Applicable.

**Table:** 

Incidence of Tumors in Females						
Tumor type	Vehicle control (n = 25)	Pretomanid (mg/kg/day)			Positive control (n = 4)	HCR (n = 25-30)
		10 (n = 25)	30 (n = 25)	80 (n = 25)		
**Lung tumors**						
Adenoma						
Single	4%	0%	4%	0%	0%	Up to 24%
Multiple	0%	0%	0%	0%	100%*	Up to 8%
Carcinoma	4%	0%	0%	8%	0%	Up to 12%
Combined	8%	0%	4%	8%	100%*	NA
**Hemangiosarcoma**						
Spleen	4%	0%	0%	8%		0-16%
Multicentric**	0%	4%	0%	0%		0-4%
Uterus	0%	4%	0%	0%		0-8%
Vagina	0%	0%	4%	0%		0-4%
Combined	4%	8%	4%	8%		NA
**Other tumors**						
Multicentric lymphoma	0%	0%	0%	4%		0-8%
Harderian gland adenoma	0%	0%	8%	0%		0-16%
Harderian gland carcinoma	4%	4%	0%	8%		0-8%
Thymus, thymoma	0%	4%	0%	0%		0-12%
Urethra papilloma	0%	0%	0%	4%		NPO

*Statistically significant (*P* < 0.01).

**Includes abdominal skin and thoracic wall.

Vehicle = 0.5% sodium carboxymethylcellulose/0.1% Tween 80, in deionized water.

Positive control = Three IP injections of 1000 mg/kg/day urethane in 0.9% saline.

HCR = BioReliance Historical Control Range for control mice (25-30 animals/sex/group).

NA = Not Applicable.

NPO = Not previously observed.

#### Hemangiosarcoma

Pretomanid did not significantly increase the incidence of hemangiosarcoma in any single organ or in multiple organs at any dose level (Table 8). The incidence of hemangiosarcoma was similar in all groups and was within the historical control range at the laboratory (for 25-30/animals/sex/group).

#### Other tumors

Pretomanid did not significantly increase the incidence of non-vascular and non-pulmonary tumors at any dose level. Such tumors were infrequent, and the incidence of each was within historical control ranges at the laboratory (for 25-30/animals/sex/group). One female at 80 mg/kg/day had a urethral papilloma, which has not been previously observed at BioReliance. This was considered to be an incidental and spontaneous tumor unrelated to Pretomanid.

### Non-Neoplastic Microscopic Findings

Pretomanid did not produce non-neoplastic microscopic findings at any dose level. The non-neoplastic findings noted in this study were considered to be spontaneous or incidental, and/or their incidence was similar across the dose groups.

### Systemic Exposure to Pretomanid and its M50 Metabolite

On Day 14, mice given pretomanid had plasma exposure to both pretomanid and its metabolite (M50) at all dose levels in both sexes. Exposure to pretomanid was more than 10 times greater than exposure to the M50 metabolite (Table 9).

**Table 9.** Toxicokinetic Parameters.

Sex	Dose (mg/kg/day)	Pretomanid		M50 metabolite		
		C_max_ (μg/mL)	AUC_0-24_ (μg.hr/mL)	C_max_ (μg/mL)	AUC_0-24_ (μg.hr/mL)	% AUC (M50/parent)
Male	5	1.8	13.7	0.09	1.19	8.7
	15	4.9	50.8	0.21	2.65	5.2
	40	10.4	112	0.32	4.57	4.1
Female	10	2.9	24.9	0.16	1.80	7.2
	30	6.9	87.5	0.34	4.87	5.6
	80	16.2	186	0.68	10.20	5.5

C_max_ = maximum plasma concentration.

AUC _0-24_ = area under the plasma concentration vs time curve over 24 hours postdose.

M50 - hydroxy imidazole metabolite of pretomanid.

## Discussion

Based on the expected response of lung tumors in the positive control animals, the high survival rates in all groups and the accuracy of the pretomanid concentrations in the formulations, the 26-week mouse carcinogenicity study was considered valid. Based on the absence of any dose-related increase in tumor incidence, pretomanid was considered negative for carcinogenicity in the TG.rasH2 mouse model.

There is a significant amount of literature on the mutagenicity of heterocylic nitro compounds. With few exceptions, they tend to be positive in Ames assays.^18^ The negative results with certain compounds have been attributed to structural features making them less reactive. Experiments using bacteria with reduced nitroreductase activity have also shown reduced or negative mutagenicity,^19^ suggesting that mutagenic activity may be due to production of reactive metabolites by reductase enzymes in bacteria. Such enzymes are not highly expressed in mammalian cells, suggesting that bacterial mutagenicity assays may overpredict the risk of mutagenic potential in humans. It is also believed that anaerobic environments enable genotoxic responses from these compounds through free radical production.

Much fewer data are available on the clastogenicity of heterocyclic nitro compounds *in vitro* from micronucleus assays or chromosomal aberration assays. Overall, regarding in vitro clastogenicity in mammalian cells, most heterocyclic nitro compounds are negative for clastogenicity in vitro, but there are some positive results or equivocal results.^20,21^ Presumably, for this class of compounds, positive results in mammalian cells should be considered more predictive for human risk than positive results in bacteria.

Pretomanid was negative for both mutagenic and clastogenic potential in a standard battery of valid, GLP-compliant, *in vitro* and *in vivo* studies. These included a bacterial reverse mutation (Ames) assay, an *in vitro* chromosomal aberration assay in CHO cells, and a bone marrow micronucleus assay in mice given single oral doses of pretomanid. Pretomanid also was negative for genotoxicity in a non-GLP *in vitro* mouse lymphoma assay at concentrations up to 1000 μg/mL (data not shown).

In contrast, the hydroxy imidazole metabolite of pretomanid, designated as M50, which is present in plasma of rats, monkeys, and humans given pretomanid, was positive for mutagenic potential in a bacterial reverse mutation assay. Metabolite M50 was not directly evaluated for clastogenic potential. However, it is likely that this metabolite was formed by the rat liver S9 mix used as metabolic activation during in vitro clastogenicity assays with pretomanid in CHO cells and mouse lymphoma cells, because the metabolite is formed by rats *in vivo*. It also is likely that the metabolite was present in mice given oral doses of pretomanid in the bone marrow micronucleus study, since it was formed by mice in the carcinogenicity study. However, the amount of metabolite M50 formed in these assays is unknown.

Taken together the results of the genotoxicity studies support the conclusion that pretomanid itself does not present a genotoxic hazard to human subjects and therefore is unlikely to pose a carcinogenic hazard, even though metabolite M50 is mutagenic in bacterial reverse mutation assays. However, because pretomanid is recommended for administration to patients for up to 6 months, its carcinogenic potential was evaluated in two studies. The current publication describes a 26-week carcinogenicity study in Tg.rasH2 mice. A 104-week carcinogenicity study in rats was in progress at the time this paper was written.

The toxicokinetic data from this study showed that the mice were exposed systemically to both pretomanid and the M50 metabolite, but the M50 was quite low relative to the parent compound. The AUC_0-24_ of the M50 metabolite was between 4 and 9% of the AUC_0-24_ of the parent compound in mice. The M50 metabolite was not measured during clinical trials, however, a mass balance study in humans using ^14^C-labelled pretomanid showed the M50 metabolite was ∼6% of the parent compound (unpublished data). Thus, estimating the AUC_0-24_ of M50 based on 6% of the pretomanid therapeutic AUC_0-24_ would yield an estimated AUC_0-24_ of ∼3 μg.hr/mL. Comparing systemic exposure in mice measured in this carcinogenicity study to human systemic exposures, the gender averaged mouse AUC_0-24_ of pretomanid at the high dose was 149 μg.hr/mL, approximately 3x the human AUC_0-24_ of 50.9 μg.hr/mL measured at a dose of 200 mg/day in a clinical study (ClinTrials.gov Identifier: NCT02333799).^22^ The mean M50 AUC_0-24_ measured at the highest dose in the carcinogenicity study was 4.6 g.hr/mL in males and 10.2 μg hr/mL in females. These exposures are approximately 1.5X and 3X the estimated human AUC_0-24_ of the M50 at the recommended clinical dose of 200 mg/day.

Most drugs containing nitroaromatic groups that have been tested in carcinogenicity studies have shown some evidence of carcinogenicity, but the compounds vary in pattern and strain sensitivity (see Table 10).

**Table 10.** Genotoxicity and Carcinogenicity Profile of Nitroaromatic Drugs.

Drug	Indication	Mice	Rats	Genotoxicity
Metronidazole	Trichamoniasis, Serious bacterial infections	Carcinogenic	Carcinogenic	Positive in vitro, negative in vivo
Nitrofurantion	Bacterial urinary tract infections	Not carcinogenic in Swiss or BDF mice, carcinogenic in female B6C3F1 mice	Not carcinogenic in Holtzman rats or Sprague-Dawley rats, carcinogenic in male F344/N rats	Positive in vitro, negative in vivo
Benznidazole	Trypanosomiasis	Not tested	Not tested	Positive in vitro and in vivo
Entacapone	Parkinsons disease	Not carcinogenic	Carcinogenic	Positive in vitro, negative in vivo

*Source: Drug Prescribing information.

There are several possible reasons why exposure to M50 did not cause an increase in tumor incidence in Tg.rasH2 mice. Possibly exposure to metabolite M50 did not reach a level that exceeded the capacity of DNA repair and other cellular defense mechanisms. Another possibility is that the way in which DNA is packaged in mammalian cells protects it from damage by metabolite M50, compared to the relatively naked DNA in bacterial cells. These possibilities are not mutually exclusive.

## Conclusions

As a class, heterocyclic nitro compounds are considered potentially mutagenic and carcinogenic by way of metabolic activation that can produce reactive oxygen species (ROS) and free radicals. Most such compounds are mutagenic in bacteria, and several also have been positive in rodent carcinogenicity studies. Pretomanid is an exception to these generalizations, in that it was negative for genotoxicity in a battery of in vitro and in vivo studies, including a bacterial mutagenicity assay and negative for carcinogenicity in a 26-week study in Tg.rasH2 mice. Therefore, the safety profile of pretomanid compares favorably with other drugs containing nitroaromatic moieties.
